# Combining in vivo and in vitro approaches to better understand host‐pathogen nutritional interactions

**DOI:** 10.1111/1365-2656.70000

**Published:** 2025-02-07

**Authors:** Robert Holdbrook, Catherine E. Reavey, Joanna L. Randall, Awawing A. Andongma, Yamini Tummala, Annabel Rice, Stephen J. Simpson, Judith A. Smith, Sheena C. Cotter, Kenneth Wilson

**Affiliations:** ^1^ Lancaster Environment Centre Lancaster University Lancaster UK; ^2^ Charles Perkins Centre The University of Sydney Sydney New South Wales Australia; ^3^ School of Forensic and Applied Sciences University of Central Lancashire Preston UK; ^4^ School of Life Sciences University of Lincoln Lincoln UK

**Keywords:** immunity, nutribloods, nutritional geometry, pathogenic bacteria, *Spodoptera*, *Xenorhabdus*

## Abstract

1. Nutrition often shapes the outcome of host–parasite interactions, however understanding the mechanisms by which this occurs is often confounded by the intimate nature of the association and by the fact that the host and parasite may compete for the same limiting nutrients. One way of disentangling this interaction is to combine in vivo and in vitro approaches.
2. Here, we explore the role of host nutrition in determining the outcome of infections using a model insect‐bacterium system: the cotton leafworm *Spodoptera littoralis* and the blood‐borne bacterium *Xenorhabdus nematophila*.
3. *Spodoptera littoralis* larvae were reared on one of a series of 20 chemically‐defined diets ranging in their protein: carbohydrate (P:C) ratio and caloric density. They were then challenged with either a fixed dose of *X. nematophila* cells (live or dead) or were sham‐injected. Survivorship of larvae challenged with live bacterial cells was strongly dependent on the protein levels of the diet, with mortality being highest on low‐protein diets. This trend was reflected in the bacterial growth rate in vivo, which peaked in larvae fed low‐protein diets.
4. To determine whether in vivo bacterial growth rates were driven by the direct effects of blood nutrients or by the indirect effects of the host immune response, we used 20 synthetic haemolymphs (‘*nutribloods*’) that mimicked the nutritional content of host blood. In vitro bacterial growth rate was negatively impacted by the protein content of the nutribloods, replicating the patterns seen in vivo and suggesting that nutrient availability and not host immunity was driving the interaction.
5. By comparing standardized bacterial growth rates in vivo and in vitro, we conclude that the outcome of this host–parasite interaction is largely driven by the ‘bottom‐up’ effects of nutrients on bacterial growth, rather than by the ‘top‐down’ effects of nutrients on host‐mediated immune responses. The outcome of host–parasite interactions is typically assumed to be strongly determined by the host immune response. The direct effects of nutrition have been underexplored and may have broad consequences for host–parasite interactions across taxa.

## Graphical abstract

This is one of the first studies that use a combination of in vitro and in vivo approaches to tease apart the importance of the ‘top‐down’ and ‘bottom‐up’ effects of host nutrition in determining the outcome of host‐pathogen interactions.

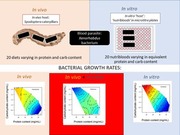

## 1 INTRODUCTION

Nutritional immunology explores the role of nutrient availability in the delicate balance between hosts and their parasites (Pernice et al., 2014; Ponton et al., 2013; Povey et al., 2014). Much of this research has focused on the effects of nutrients on host immune function and/or on the outcome of an infection, with host fitness often being positively correlated with elevated levels of host immunity (Ponton et al., 2013; Schmid‐Hempel, 2021; Wilson, Fenton, & Tompkins, 2019). What is often overlooked in these studies is the effects of host nutrition on parasite establishment and proliferation. This is an important knowledge gap because parasites are usually dependent on their hosts for nutritional resources and both the host and its parasite may compete for the same limiting nutrients. Thus, the outcome of an infection may be determined primarily by the effects of host nutrition on ‘top‐down’ (e.g. immunological) processes directed at the parasite, ‘bottom‐up’ effects of specific nutrients on parasite population growth, or a combination of the two (Griffiths et al., 2015; Haydon et al., 2003; Metcalf et al., 2011; Mideo & Reece, 2012; Moore et al., 2018; Ramiro et al., 2016). Disentangling the relative importance of top‐down and bottom‐up regulation of parasites is difficult due to the intimate nature of the association, but it is possible by combining in vivo and in vitro approaches in a tractable system.

Here, we use as a model system for teasing apart a host–parasite interaction in a generalist caterpillar host, the cotton leafworm *Spodoptera littoralis* and its parasite *Xenorhabdus nematophila*, an extra‐cellular gram‐negative bacterium. The bacterium has a mutualistic association with the entomopathogenic nematode *Steinernema carpocapsae*, which vectors the bacterium into insect hosts via the cuticle or orifices such as the mouth and anus. Importantly, *X. nematophila* is also able to kill its insect host without the nematode when it is injected directly into the insect haemocoel (Wilson et al., 2020). *S. littoralis* is a useful host because it is relatively easy to culture in the laboratory, it is a generalist feeder and large amounts of haemolymph can be extracted from a single individual, allowing multiple blood tests to be undertaken.

Nutritional geometry (NG) is a state‐space nutritional modelling approach that is aimed at determining the effects of multiple nutrients on an organism's behaviour and fitness. A previous study developed 20 chemically‐defined diets systematically varying in their concentration and balance of two key macronutrients, proteins and carbohydrates (Cotter et al., 2011). These 20 diets reflect the variation in these macronutrients that *S. littoralis* would naturally encounter in its environment (Figure S1; Scott Brown et al., 2002; Wilson, Ruiz, & Davidowitz, 2019). Using these diets, it has been shown that some aspects of *S. littoralis* immune responses are heightened in a high‐protein environment (Cotter et al., 2011, 2019; Lee et al., 2006), demonstrating the potential for top‐down (immunological) effects on bacterial growth. In another study (Holdbrook, Andongma, et al., 2024), this approach was used to establish the effects of host nutrition on the insect haemolymph (blood) nutrient pool for insects feeding on the same 20 chemically‐defined diets. This established that whilst carbohydrates in the haemolymph are generally tightly regulated, haemolymph protein concentration tends to increase with the amount of protein eaten (Holdbrook, Andongma, et al., 2024). In a subsequent study (Holdbrook, Randall, et al., 2024), these data were used to generate 20 synthetic haemolymphs (‘*nutribloods*’) that mimicked the nutritional profile of the real haemolymphs of caterpillars fed the 20 chemically‐defined diets. This revealed that the in vitro growth of *X. nematophila* (in the absence of host immune defences) increased with the amount of carbohydrate in the nutriblood and decreased with the amount of protein, suggesting potential bottom‐up effects of nutrition on bacterial performance.

Here, we combine these in vitro nutriblood results in our model host‐pathogen system with in vivo bacterial dynamics to tease apart the relative importance of top‐down and bottom‐up effects of host nutrition in determining the outcome of infections by *X. nematophila* in *S. littoralis*. The work builds on an earlier study that explored this interaction using just six chemically‐defined diets covering a limited range of nutrient space (Wilson et al., 2020). In the present study, we combine in vitro and in vivo experiments using 20 chemically‐defined diets and nutribloods to test the robustness of this finding and to statistically compare bacterial growth in the two settings. We argue that this combination of in vitro and in vivo approaches could be used with other systems to test similar questions, as well as more broadly to look at how host nutrition affects competition between competing pathogens and symbionts.

## 2 METHODS

### 2.1 Cultures

#### 2.1.1 Insect culture

The *Spodoptera littoralis* culture was founded in 2002 from eggs collected from Egypt. It was maintained using single‐pair matings of over 150 pairs per generation to reduce inbreeding. For experiments, larvae were collected in the 2nd instar and reared singly on a semi‐artificial wheatgerm‐based diet based on Hunter et al. (1984) until the start of the final instar (6th). Larvae were kept in 25 mL polypots at 27°C under a 12:12 light: dark regime.

#### 2.1.2 Bacterial culture

Bacteria were originally supplied by the laboratory of Givaudan and colleagues (Montpellier University, France; *X. nematophila* F1D3 GFP labelled). It was maintained on nutrient agar at 4°C and stored in liquid culture at −80°C (1:1 nutrient broth culture: glycerol). Bacteria were used to infect 6th instar *S. littoralis* larvae to maintain virulence and single colonies grown from haemolymph‐smeared NBTA agar plates were then grown in sterile nutrient broth for 24 h at 28°C shaking at 150 rpm. Stocks were made by mixing 500 μL of liquid culture with glycerol at a 1:1 ratio and stored again at −80°C. Prior to experiments, bacteria were revived from the frozen stores: 100 μL of frozen culture was added to 10 mL nutrient broth, which was then incubated for 16 h at 28°C shaking at 150 rpm.

### 2.2 In vivo experiments

#### 2.2.1 Bacterial culture

The methods for the in vivo experiments are based on those of Wilson et al. (2020). In brief, on the day of the bacterial challenge, the bacterial stock was sub‐cultured in nutrient broth and placed in a shaker‐incubator for *c*. 4 h to ensure that the bacteria were in the log phase. Following this, the concentration of bacterial cells was quantified using a fluorescence microscope in a serial dilution of nutrient broth using a haemocytometer with improved Neubauer ruling. The remaining culture was further diluted with nutrient broth to the appropriate concentration required for the bacterial challenge. Half of the culture was then autoclaved to use as a ‘dead bacteria’ control group.

#### 2.2.2 Experimental design

Four hundred larvae were reared to the start of the 6th instar on a semi‐artificial wheat germ‐based diet. Within 24 h of moulting into the final instar, the larvae were divided into 20 groups (*n* = 20 larvae) and placed singly onto one of twenty diets differing in dietary attributes (Table S1). Between 1.8 and 2.1 g of the chemically‐defined diets were placed in 90 mm diameter Petri dishes and the larvae were housed in this manner throughout the experiment with the diet replaced every 24 h. Within each diet, 10 caterpillars were allocated to a ‘live bacteria challenged’ group (henceforth live‐infected), 5 caterpillars were assigned to a ‘dead bacteria challenged’ group (henceforth dead‐infected) and 5 caterpillars were allocated to a ‘sham‐challenged’ group (henceforth sham‐infected). For the live‐infected caterpillars, the bacterial dose used was 1272 *X. nematophila* cells/mL nutrient broth. This dose was established from pilot experiments to determine the LD_50_ (Wilson et al., 2020). The same dose was used for the dead‐infected challenge, albeit the challenge would consist of cell debris as a result of autoclaving. The sham‐infected caterpillars were injected with autoclaved nutrient broth only.

Following 24 h on the assigned diets, each of the 400 caterpillars was injected with the appropriate treatment; 5 μL of live *X. nematophila*, 5 μL of heat‐killed *X. nematophila* or 5 μL of autoclaved nutrient broth. Injections were carried out using a Hamilton Syringe in a micro‐injector. The syringe was sterilized in ethanol before each injection and the challenge was applied to the left proleg nearest to the head. The time of injection was recorded due to the need to control for the length of time between injection of the first and last individuals (4.5 h). Injections were randomized across treatments.

After the challenge, haemolymph samples were obtained from all caterpillars at approximately 20 h post‐infection. Samples were obtained by piercing the cuticle next to the first proleg near the head with a sterile needle and allowing released haemolymph to bleed directly into an Eppendorf tube. Haemolymph samples from all the live‐injected caterpillars were plated out to determine bacterial growth (*n* = 200). One of each of the 5 caterpillars for both the dead‐infected and sham‐infected caterpillars within each dietary treatment was plated out to ensure no bacterial contamination had occurred (*n* = 40). Immediately after obtaining the haemolymph, the relevant samples were diluted in pH 7.4 phosphate‐buffered saline (PBS; 10 μL of haemolymph placed in 90 μL of PBS and so on through the dilution series) down to 10^−7^ at intervals of 10^−1^. The dilution series was plated onto NBTL agar plates (20 μL per 1/4 agar plate) containing bromothymol blue and triphenyltetrazolium chloride and incubated at 28°C. *Xenorhabdus* colonies appear deep blue on these NBTL agar plates, whereas most other bacterial species appear yellow or red, allowing contaminants to be identified. Although most of the colonies were visible at 24 h, there were some slow‐growing colonies that were not visible until 48 h. Following the incubation period at 28°C, the CFUs were counted for each sample and then the CFU/mL haemolymph was determined based on the dilution factor at which colonies could be reliably counted.

Fresh diet was provided in clean 90 mm diameter Petri dishes every 24 h up to 72 h (48 h post‐challenge). Ninety‐six hours after moulting into the 6th instar, the larvae had either pupated or were placed in pots of semi‐artificial diet until death or pupation. All caterpillars were monitored for death throughout the day of sampling and every day after until pupation or death.

### 2.3 In vitro growth experiments

#### 2.3.1 Synthetic haemolymphs: Nutribloods

The full methods for generating the 20 nutribloods are outlined in (Holdbrook, Randall, et al., 2024). In brief, the design was based on the nutritional composition of *S. littoralis* fed on the 20 chemically‐defined diets using a combination of (ultra‐) high‐performance liquid chromatography (uHPLC and HPLC) and spectroscopic methods (Holdbrook, Andongma, et al., 2024), using Grace's insect medium (Sigma Aldrich G8142) as a source of minerals and vitamins (Tables 1–3 in Holdbrook, Randall, et al., 2024).

#### 2.3.2 Preparation of bacteria

The methods for preparing the bacteria are outlined in full (Holdbrook, Randall, et al., 2024). In brief, bacteria were revived from frozen liquid stores and sub‐cultured in nutrient broth before being incubated for 4 h to reach the log phase of growth. The bacterial cells were washed in PBS to avoid the transfer of nutrients from nutrient broth into the growth media, following (Crawford et al., 2012). A 1 mL sample was used to generate a dilution series in GIM‐saline from which the total cell count was determined using a haemocytometer with improved Neubauer ruling. The remaining culture was then diluted in the GIM‐saline solution to make the final starting concentration in each treatment 1 × 10^6^ cells/mL. (Sprouffske & Wagner, 2016).

#### 2.3.3 Bacterial growth assays

Bacterial cell growth was quantified using a SpectraMax Plus microtiter plate reader (Molecular Devices) with SoftMax Pro software (Wilson et al., 2020). Each plate contained 180 μL of one of the 20 nutribloods in quadruplets. The turbidity at 600 nm was determined every 10 min for 30 h and the plate was shaken for 30 s before each measurement.

### 2.4 Data analysis

All data analyses were performed using the R statistical software (v4.3.0; R Core Team, 2023) and RStudio (version 2023.6.0.421; Posit team, 2023). The in vitro bacterial growth kinetics were quantified using the *Growthcurver* package (v0.3.1; Sprouffske & Wagner, 2016), with the maximum optical density (OD) at 600 nm. Insect survivorship post‐challenge until death (in the larval, pupal or moth stage) was analysed by fitting a parametric survival regression model, using the *survreg* function in the survival package (v3.5.5; Therneau, 2024). Speed of death was quantified as 1/time to death (h). Both in vivo and in vitro data were analysed using generalized additive models (GAMs) in the *mgcv* package (v1.8.42) (Wood, 2017) in conjunction with thin‐plate spline plots using the *fields* package (v14.1; Nychka et al., 2017), following (Cotter et al., 2011). To account for variation in haemolymph nutrient concentrations, data were standardized using the mean (μ) and standard deviation (σ), as per Cotter et al. (2011). An information theoretic approach was taken for analysis (Whittingham et al., 2006), which allows a selection of multiple candidate models to be simultaneously compared based on corrected Akaike information criteria (AICc; Burnham & Anderson, 2004). This was carried out using the *MuMIn* package (v1.47.5; Bartoń, 2023) in R which, when combined with the *mgcv* package, ranks models based on AICc values. The specific analyses varied, however, they all included a ‘*Null* model’, which provided a baseline measure of variation. Models were considered to be indistinguishable where ΔAICc <2. If more than one model met this criterion, the selected model was the one with the lowest numbers of parameters, that is, the simplest model.

## 3 RESULTS

### 3.1 Host survivorship in relation to bacterial challenge status

**FIGURE 1:**
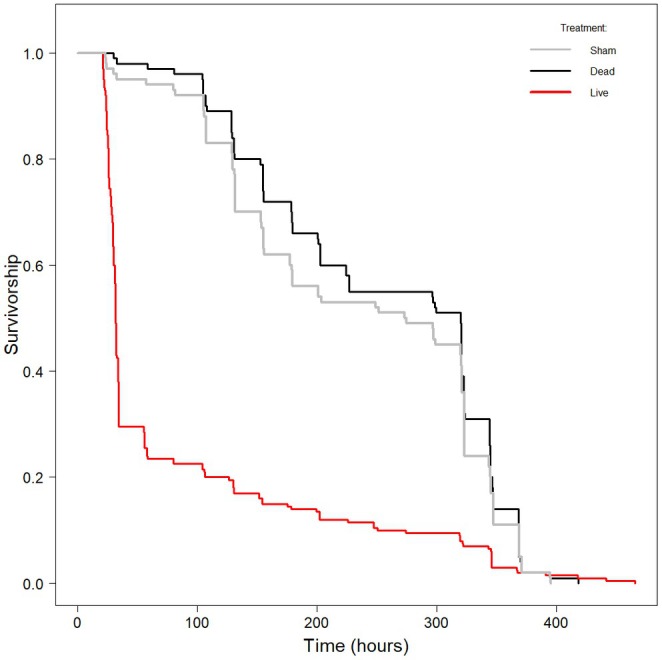
Survivorship curves (time‐specific survival) for larvae in the three treatment groups. None of the larvae in the sham or dead‐infected groups died of *Xenorhabdus nematophila* infection. Insects were monitored until death (whether as larvae, pupae or moths), with curves censored at the timepoint where no live individuals remained. [Correction added on 13 February 2025, after first online publication: The colour of the lines in Figure 1 have been corrected.]

Overall larval mortality was 80% (*n* = 160/200) in insects challenged with live bacteria, 6% (*n* = 6/100) in those challenged with dead bacteria and 11% (11/100) in larvae that were sham‐challenged; only insects in the live‐challenged group died of *X. nematophila* infection. Live‐challenged larvae lived for a median time (IQR) of 31.7 h (26.3, 57.6), whereas those in the dead‐challenged and sham‐challenged groups lived, on average, for another week before either dying or pupating: 320.3 h (154.9, 344.7) and 274.2 h (131.4, 323.5), respectively (Figure 1). Survival analysis indicated that there was a marked difference in the survivorships of insects in the three treatment groups overall (χ^2^_2_ = 140.87, *p* < 0.0001), with the live‐challenged insects suffering higher mortality rates than the two control groups (*z* = −10.29, *p* < 0.0001), which did not differ in their survivorships (*z* = −0.63, *p* = 0.53).

### 3.2 Host speed of death in relation to host diet

Given the very different mortality rates in the live‐challenged larvae compared to those in the two control groups, the effect of diet on standardized speed of death (see Section 2 for calculation) was compared across these two groups separately. In the two control groups, diet had little effect on the speed of death, explaining less than 5% of the variation (Table 1), regardless of whether the larvae had been challenged with dead bacteria or sham‐challenged (Figure S2A,B).

**TABLE 1:** GAMs explaining speed of death in relation to the nutritional attributes of the host diet for larvae in the two control groups.

(a) Model	Parameters	AIC_c_	Δ AIC_c_	*w*	*r* ^2^
3. Protein + Carb	3	228.5	0.00	0.233	0.044
4. Protein * Carb	4	228.6	0.06	0.226	0.022
2. Carb	2	228.7	0.19	0.212	0.016
**0. No diet attributes**	**1**	**229.1**	**0.55**	**0.177**	**0.009**
1. Protein	2	229.4	0.84	0.153	0.013

(b) Parametric terms	*b*	SE	*t*	*p*
Intercept	−0.75229	0.04246	−17.717	<0.0001
Sham‐challenged	0.09873	0.06005	1.644	0.102

*Note*: (a) Table of candidate models. AICc = corrected Akaike information criteria values; Δ AICc = difference in AICc values between the best model (lowest AICc) and the given model; w = Akaike weights; *r* ^2^ = pseudo‐*r* ^2^ for the model. The five alternative Diet attributes listed in the first column are described in Table S1. The dependent variable in these models is the speed of death (1/time taken to die, h), standardized to have a mean of zero and a standard deviation of unity for all larvae (see Section 2). Analysis was restricted to those larvae that were challenged with dead bacteria or sham‐challenged. (b) Parametric terms of the top model (model 0). The model in bold type is the top model.

The z‐value range in this plot is fixed such that low values appear dark blue and high values are increasingly warmer colours.

A previous study (Wilson et al., 2020) suggested that insects challenged with live bacteria comprise three categories of individuals: those that succumb to the bacteria (and die if/when the bacterial load exceeds some critical threshold); those that successfully control the nascent bacterial population and survive infection; and those that stochastically did not receive a sufficiently large dose of cells for the bacterial population to establish. In practice, it is difficult to distinguish between these latter two categories, but we can ask whether the nutritional properties of the diet affect differently larvae that survived a live bacterial challenge (whether infected or not) versus those that did not survive (and certainly did host a growing bacterial population). This revealed that diet did not affect the speed of death of those individuals that survived the bacterial challenge (Table 2; Figure S2C), but larvae succumbing to bacterial infection lived longer if they were fed on protein‐rich diets (Table 2; Figure S2D). Given that diet only appears to affect the speed of death of larvae dying of *X. nematophila* infection, all further analyses presented here are restricted to this category of insects.

**TABLE 2:** GAMs explaining speed of death in relation to the nutritional attributes of the host diet for larvae in the live bacteria challenged group.

(a) Model	Parameters	AIC_c_	Δ AIC_c_	*w*	*r* ^2^
3. Protein + Carb	3	245.9	0.00	0.450	0.770
**1. Protein**	**2**	**246.0**	**0.10**	**0.427**	**0.769**
4. Protein * Carb	4	248.5	2.60	0.122	0.770
0. No diet attributes	1	309.1	63.19	0.000	0.676
2. Carb	2	309.1	63.20	0.000	0.676

(b) Parametric terms	*b*	SE	*t*	*p*
Intercept	−0.79533	0.06933	−11.47	<0.0001
Died	1.89370	0.07760	24.40	<0.0001

(c) Smoothed terms	edf	Ref.df	*F*	*p*
Protein/Survived	0.0001	4	0.000	0.6030
Protein/Died	2.9191	4	19.82	<0.0001

*Note*: (a) Table of candidate models. AICc = corrected Akaike Information Criteria values; Δ AICc = difference in AICc values between the best model (lowest AICc) and the given model; *w* = Akaike weights; *r* ^2^ = pseudo‐*r* ^2^ for the model. The five alternative Diet attributes listed in the first column are described in Table S1. The dependent variable in these models is the speed of death (1/time taken to die, h), standardized to have a mean of zero and a standard deviation of unity for all larvae (see Section 2). Analysis was restricted to those larvae that were challenged with live bacteria and either died or survived. (b) Parametric terms and (c) Smoothed terms of the top model (model 1). The model in bold type is the top model.

### 3.3 In vivo bacterial growth rate in relation to host diet macronutrient composition

**FIGURE 2:**
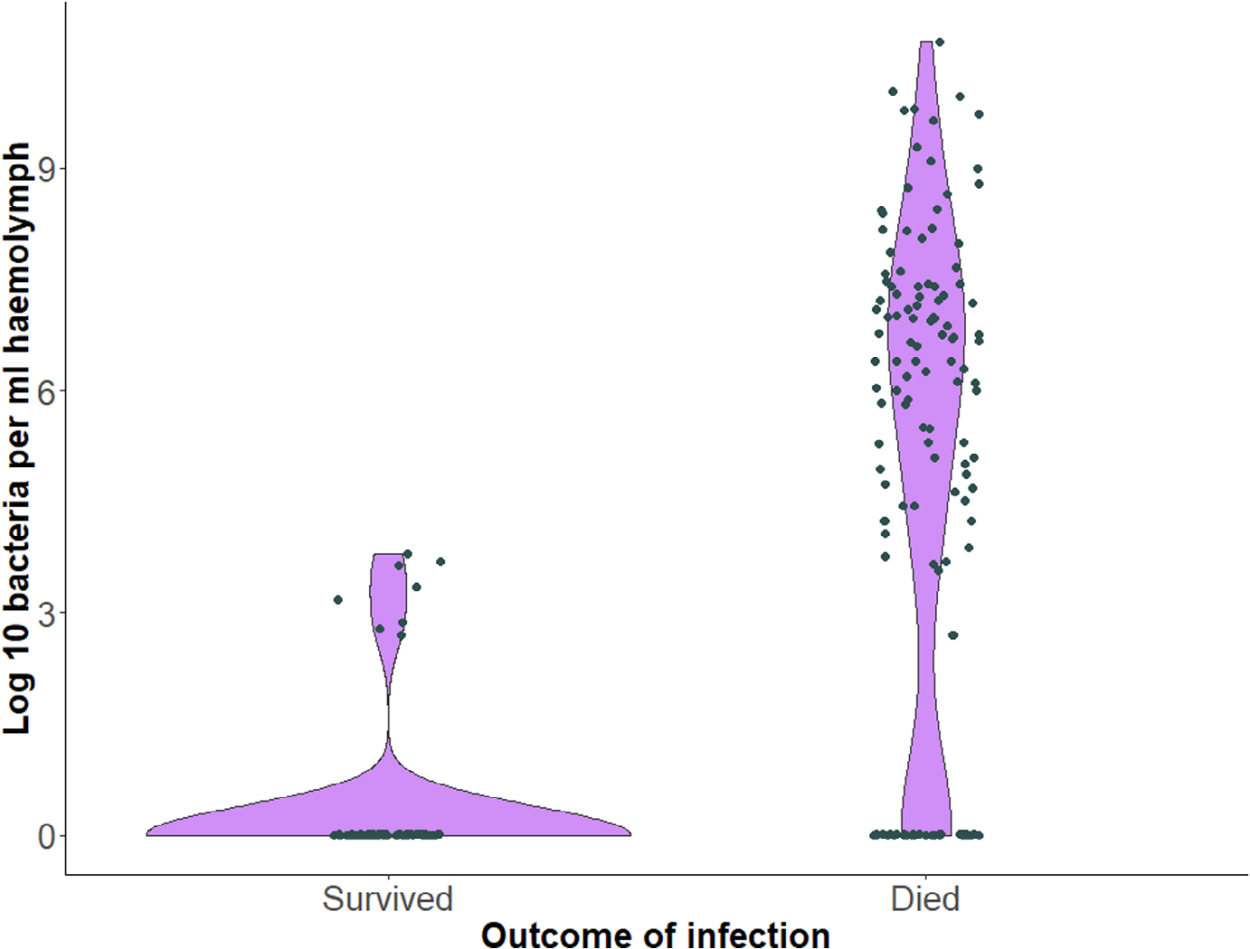
Violin plot depicting the bacterial loads of larvae at sampling with respect to whether they survived infection or died.

Bacterial counts in sampled haemolymph ~20 hours post‐infection confirmed that larvae in the two control groups were free of *X. nematophila* infection. It also showed that, at the point of sampling, larvae in the live‐challenge group harboured an average of around 10^4^ CFU/mL, but with most survivors (*n* = 30) harbouring no bacteria at sampling and with the remaining survivors averaging around 10^3^ CFU/mL (range = 5 × 10^2^–5 × 10^3^; *n* = 8). In contrast, those dying of *X. nematophila* infection averaged 10^6^ CFU/mL, with a substantial number of larvae (*n* = 31) hosting no culturable *X. nematophila* at the point of sampling and the remainder averaging 10^6^ CFU/mL (range = 5 × 10^2^–5 × 10^10^; *n* = 97; Figure 2).

**FIGURE 3:**
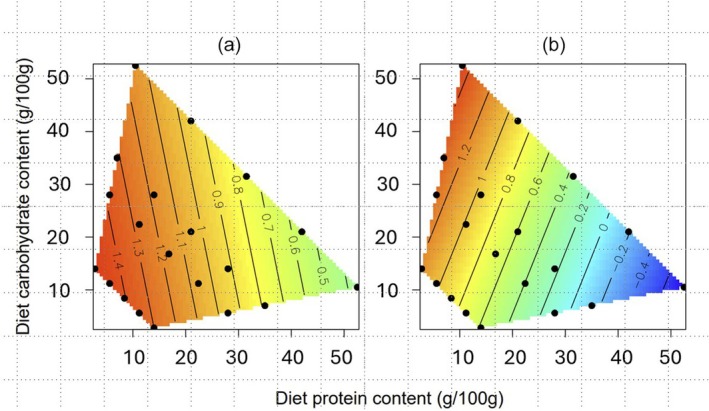
Effects of host diet on standardized speed of death and bacterial growth rate in *Xenorhabdus nematophila*‐challenged insects. Effects of protein and carbohydrate in host diet (g/100 g) on (a) standardized speed of death (1/lifespan, h) and (b) in vivo bacterial growth rate in larvae dying of *X. nematophila* infection based on log10(CFU/mL). Both panels have a common z‐limit. The hotter the colour (reds, oranges), the faster the speed of death and bacterial growth rate.

As indicated above, larvae succumbing to bacterial infection lived longer if they were fed on protein‐rich diets (Figure 3a). The bacterial load at sampling for larvae that would subsequently die of infection (i.e. the in vivo bacterial growth rate) was also largely determined by the amount of protein in the host diet, explaining more than 30% of the variation, with little contribution from the amount of dietary carbohydrate (Figure 3b; Table 3); bacterial load markedly decreased as the protein content of the diet increased, and increased slightly as the amount of carbohydrate increased.

**TABLE 3:** GAMs explaining standardized in vivo bacterial growth rate (i.e. bacterial load at sampling) in relation to the macronutritional attributes of the host diet for larvae that died of *X. nematophila*.

(a) Model	Parameters	AIC_c_	Δ AIC_c_	*w*	*r* ^2^
**3. Protein + Carb**	**4**	**608.6**	**0.00**	**0.452**	**0.347**
4. Protein * Carb	5	608.6	0.00	0.452	0.347
1. Protein	3	611.8	3.11	0.096	0.328
2. Carb	3	653.9	45.28	0.000	0.074
0. No diet attributes	2	659.2	50.59	0.000	0.000

(b) Parametric terms	*b*	SE	*t*	*p*
Intercept	0.51206	0.06627	7.727	<0.0001

(c) Smoothed terms	edf	Ref.df	*F*	*p*
Bleeding time	0.00057	4	0.000	0.3932
Protein	0.98299	4	14.0118	<0.0001
Carb	1.38181	4	1.479	0.0153

*Note*: (a) Table of candidate models. AICc = corrected Akaike Information Criteria values; Δ AICc = difference in AICc values between the best model (lowest AICc) and the given model; *w* = Akaike weights; *r* ^2^ = pseudo‐*r* ^2^ for the model. The five alternative Diet attributes listed in the first column are described in Table S1. The dependent variable in these models is log_10_(bacterial load, CFU/mL). Analysis was restricted to those larvae that died of bacterial infection. (b) Parametric terms and (c) Smoothed terms of the top model (model 3). The model in bold type is the top model.

### 3.4 In vivo bacterial growth rate in relation to haemolymph macronutrient composition

In a previous paper (Holdbrook, Andongma, et al., 2024), the effects of host diet on the nutritional composition of the host haemolymph was explored—the environment in which *X. nematophila* would grow. To establish how the nutritional composition of *S. littoralis* haemolymph translates into bacterial growth rates, the relationship between these traits was quantified in the average haemolymph composition of larvae fed on each of the 20 diets. It should be noted that both haemolymph protein and haemolymph carbohydrate are positively correlated with the relative amount of protein and carbohydrate in the host diet, respectively: *r* (protein) = 0.685, df = 115, *p* < 0.0001; *r* (carbohydrate) = 0.739, df = 115, *p* < 0.0001. It is also pertinent to note that, in the host haemolymph, these two macronutrients are strongly negatively correlated (*r* = −0.519, df = 115, *p* < 0.0001), making it difficult to distinguish their independent effects.

**FIGURE 4:**
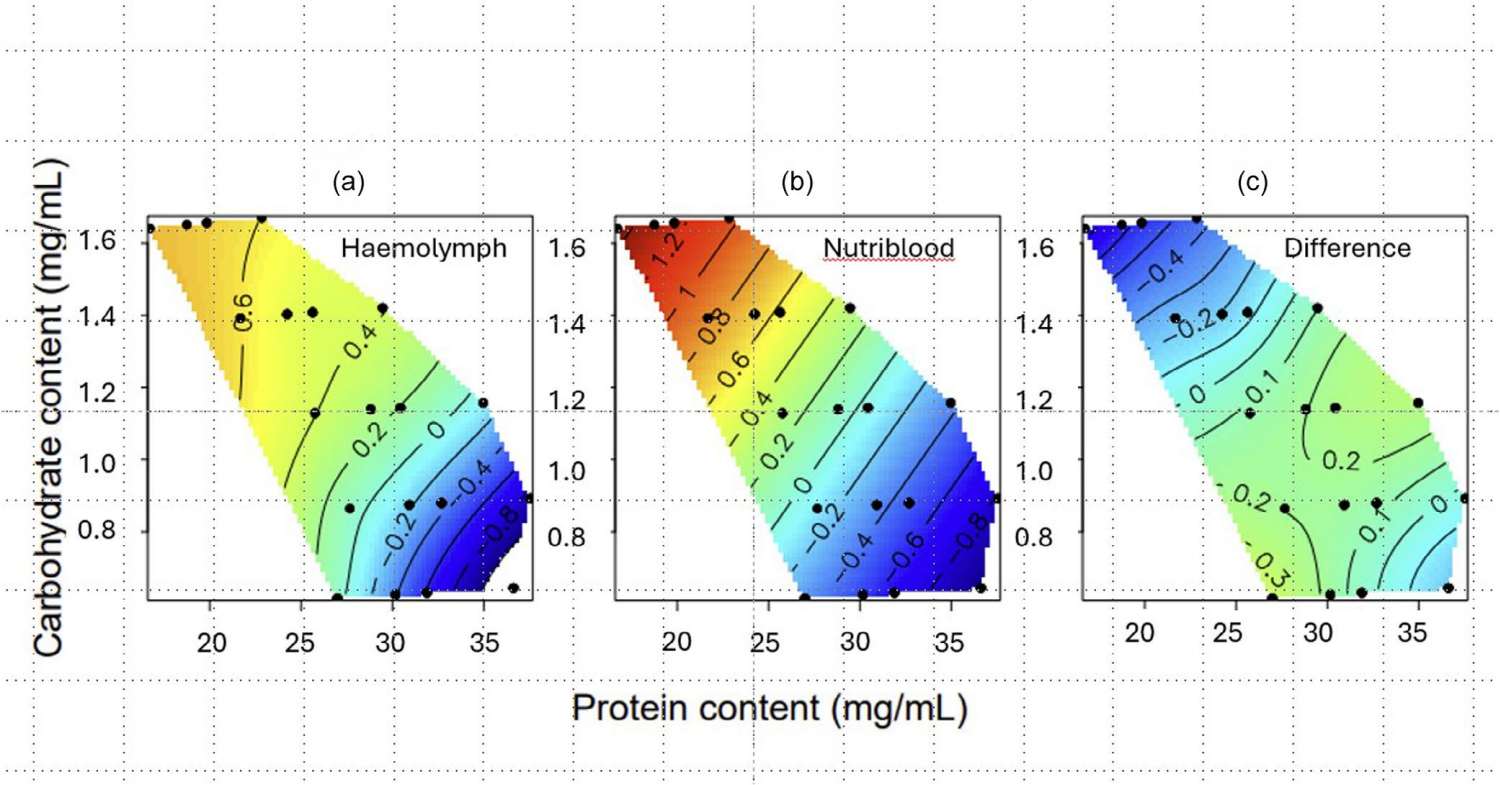
The effects of nutrients on in vitro bacterial growth rates. Relationships between (a) haemolymph macronutrients and (b) nutriblood macronutrients on standardized in vivo bacterial growth rate, as measured by culturable CFU, and standardized in vitro bacterial growth rate of *Xenorhabdus nematophila*, as measured by log maximum OD at 600 nm, respectively. (c) Difference between in vitro and in vivo standardized bacterial growth rates (in vivo minus in vitro).

As the putative amount of haemolymph carbohydrate levels increased, so too did the in vivo bacterial growth rate of dying larvae (Figure 4a, Table 4a–c, *R* ^2^ = 0.428). Given that dietary protein has previously been implicated in *X. nematophila* growth rates (Wilson et al., 2020), its higher *R* ^2^ (0.447) and that the heatmap (Figure 4a) appears to suggest a strong protein effect, we also present the outputs from the second‐best model which includes haemolymph protein and its haemolymph carbohydrate for comparison (Table 4d,e). This suggests that the in vivo bacterial growth rate is associated with both the putative amount of protein and carbohydrate in the host haemolymph, with in vivo *X. nematophila* growth increasing with haemolymph carbohydrate and decreasing with haemolymph protein, noting that these two nutrients covary in the haemolymph.

**TABLE 4:** GAMs explaining the in vivo growth rate of *X. nematophila* of dying larvae in relation to the putative levels of protein and carbohydrate in the host haemolymph at infection.

(a) Model	Parameters	AIC_c_	Δ AIC_c_	*w*	*r* ^2^
**2. Carb**	**3**	**162.6**	**0.00**	**0.404**	**0.428**
3. Protein + Carb	4	163.2	0.61	0.297	0.447
4. Protein * Carb	5	163.2	0.61	0.297	0.447
1. Protein	3	174.0	11.41	0.001	0.319
0. No diet attributes	2	175.9	13.28	0.001	0.273

(b) Parametric terms	*b*	SE	*t*	*p*
Intercept	0.5151	0.0748	6.888	<0.0001

(c) Smoothed terms	edf	Ref.df	*F*	*p*
Bleeding time	3.050	4	6.67	<0.0001
Carb	2.763	4	4.04	0.0011

(d) Parametric terms	*b*	SE	*t*	*p*
Intercept	0.5151	0.0748	6.888	<0.0001

(e) Smoothed terms	edf	Ref.df	*F*	*p*
Bleeding time	3.419	4	6.604	<0.0001
Protein	1.487	4	1.261	0.025
Carb	2.273	4	2.816	0.003

*Note*: (a) Table of candidate models. AICc = corrected Akaike Information Criteria values; Δ AICc = difference in AICc values between the best model (lowest AICc) and the given model; *w* = Akaike weights; *r* ^2^ = pseudo‐*r* ^2^ for the model. The dependent variable in these models is the CFU/mL haemolymph at the point of sampling and all models also included bleeding time as a smoothed covariate. (b) Parametric terms and (c) Smoothed terms of the top model (model 2). (d) Parametric terms and (e) Smoothed terms of the second‐top model (model 3). The model in bold type is the top model.

### 3.5 In vitro bacterial growth rate in relation to putative host diet based on synthetic haemolymphs—‘nutribloods’

When in vitro bacterial growth rate (as measured spectrophotometrically by maximum OD at 600 nm, *maxOD*) was analysed in relation to the amount of protein and carbohydrate in the nutribloods (and by extension in the host's haemolymph), this revealed that as the protein content of the haemolymph increased, so the in vitro growth rate of *X. nematophila* declined (Figure 4b; Table 5). It should be noted, however, that all four diet models explained a similar amount of variation in the bacterial growth rate (*r* ^2^ = 0.210–0.258), with the greatest amount of variation explained by a model that included both the amount of protein and the amount of carbohydrate in the nutriblood. This is due to the fact that the amount of protein and carbohydrate in the synthetic haemolymphs are strongly negatively correlated (Pearson's *r* = −0.751, df = 18, *p* = 0.0001).

**TABLE 5:** GAMs explaining the in vitro growth rate of *X. nematophila* in relation to the levels of protein and carbohydrate in the synthetic haemolymphs (‘nutribloods’).

(a) Model	Parameters	AIC_c_	Δ AIC_c_	*w*	*r* ^2^
**1. Protein**	**2**	**−196.8**	**0.00**	**0.296**	**0.228**
3. Protein + Carb	3	−196.7	0.15	0.275	0.258
4. Protein * Carb	4	−196.7	0.15	0.275	0.258
2. Carb	2	−195.5	1.32	0.153	0.210
0. No diet attributes	1	−182.8	14.01	0.000	0.000

(b) Parametric terms	*b*	SE	*t*	*p*
Intercept	0.4124	0.0057	72.07	<0.0001

(c) Smoothed terms	edf	Ref.df	*F*	*p*
Protein	0.9449	4	4.279	<0.0001

*Note*: (a) Table of candidate models. AICc = corrected Akaike Information Criteria values; Δ AICc = difference in AICc values between the best model (lowest AICc) and the given model; *w* = Akaike weights; *r* ^2^ = pseudo‐*r* ^2^ for the model. The five alternative Diet attributes listed in the first column are described in Table S1. The dependent variable in these models is the maximum OD at 600 nm. (b) Parametric terms and (c) Smoothed terms of the top model (model 1). The model in bold type is the top model.

### 3.6 In vivo and in vitro bacterial growth rate in relation to host diet

To determine how much variation in bacterial growth rate in vivo differs from that of bacteria growing in the synthetic haemolymphs, we produced a dataset that included both the standardized in vivo growth rates for larvae fed the 20 chemically‐defined diets and the standardized in vitro growth rates for bacteria grown in the 20 nutribloods mimicking the average nutritional properties of haemolymph collected from larvae feeding on the same 20 diets. We then asked how much of the relationship between bacterial growth rate and the nutritional properties of host ‘blood’ depended on whether the bacteria were growing in vivo or in vitro.

To do this, we included a dummy *Treatment* term classifying bacterial growth rate as in vivo or in vitro and in some models included this as a factor in the GAMs, as either a main effect or in interaction with the macronutrients. This revealed that the top model included both protein and carbohydrate and their interaction, as nutritional terms (*r* ^2^ = 0.327). *Treatment* did not explain any additional variation in bacterial growth rate (Table 6, Figure 4c).

**TABLE 6:** GAMs explaining variation in the in vivo and in vitro growth rate of *X. nematophila* in relation to the levels of protein and carbohydrate in the host diet.

(a) Model	Parameters	AIC_c_	Δ AIC_c_	*w*	*r* ^2^
4. Protein * Carb	**4**	**463.8**	**0.00**	**0.720**	**0.327**
4a. Protein * Carb + Treatment	5	466.0	2.22	0.237	0.323
3. Protein + Carb	3	470.2	6.42	0.029	0.298
3a. Protein + Carb + Treatment	4	472.3	8.60	0.010	0.298
4b. Protein * Carb * Treatment	8	474.7	10.91	0.003	0.293
3b. Protein + Carb * Treatment	6	478.1	14.35	0.001	0.284
1. Protein	2	479.1	15.32	0.000	0.254
1a. Protein + Treatment	3	481.2	17.48	0.000	0.250
1b. Protein * Treatment	4	483.7	19.92	0.000	0.243
2. Carb	2	484.4	20.67	0.000	0.230
2a. Carb + Treatment	3	485.5	22.80	0.000	0.226
2b. Carb * Treatment	4	490.1	26.31	0.000	0.215
0. intercept only	1	529.9	66.15	0.000	0.000
0a. Treatment	2	532.0	68.22	0.000	0.000

(b) Parametric terms	*b*	SE	*t*	*p*
Intercept	0.0000	0.0599	0.000	1.0000

(c) Smoothed terms	edf	Ref.df	*F*	*p*
Protein	0.946	4	6.863	<0.0001
Carb	0.983	4	4.545	<0.0001
Protein: Carb	2.367	2	2.667	0.0065

*Note*: (a) Table of candidate models. AICc = corrected Akaike Information Criteria values; Δ AICc = difference in AICc values between the best model (lowest AICc) and the given model; *w* = Akaike weights; *r* ^2^ = pseudo‐*r* ^2^ for the model. The five alternative haemolymph attributes listed in the first column are described in Table S1. The dependent variable in these models is the standardized bacterial growth rate (in vivo = log_10_‐bacterial load at sampling for individuals dying of *X. nematophila* infection and in vitro = max OD at 600 nm). The protein and carbohydrate levels in the blood were standardized prior to analysis. In models that included *Treatment* (i.e. in vivo or in vitro), it was treated as a parametric term. (b) Parametric terms and (c) Smoothed terms of the top model (model 3b). The model in bold type is the top model.

### 3.7 Correlation between bacterial growth in vivo and in vitro

Finally, since we have made comparisons in bacterial growth in vivo and in vitro using two different methods (CFU/mL and OD_600_, respectively), we compared the relationship between the two. Across the 20 diets and nutribloods, the in vivo and in vitro bacterial growth rates were positively correlated with each other (Pearson's *r* = 0.251, df = 203, *p* = 0.0003). These findings concur with other studies that have compared bacterial growth using both of these methods (González‐Pérez et al., 2019; Karamba & Ahmad, 2019; Kim et al., 2012; Kowalski et al., 2008).

## 4 DISCUSSION

This study aimed to combine data from in vitro and in vivo analyses of bacterial performance on 20 chemically‐defined diets to determine the relative importance of top‐down (immunological) from bottom‐up (resource) effects of host diet using as a model system a generalist caterpillar host and its extra‐cellular bacterial pathogen. It revealed that the host diet, most notably intake of dietary protein, markedly affected the rate at which the insects died of the bacterial infection. This, in turn, was determined by the effects of diet on the rate of in vivo bacterial growth, with hosts dying when the bacterial population had reached a critical threshold level (see also Wilson et al., 2020). This was largely driven by, or at least was strongly correlated with, the putative amount of protein in the insect host's haemolymph (blood). Using 20 synthetic haemolymphs (*nutribloods*) that mimicked the nutritional compositions of caterpillars feeding on each of the 20 diets (Holdbrook, Andongma, et al., 2024; Holdbrook, Randall, et al., 2024) we quantified relative bacterial performance in vitro in the absence of the host's immune system. This revealed a similar pattern to that seen in vivo, with bacterial growth rate being highest in nutribloods low in protein and high in carbohydrates. A statistical analysis comparing the bacterial performance profiles in vivo and in vitro revealed no difference between the two, consistent with the bacterial population being limited mainly by the direct effects of nutrients on bacterial growth rate (bottom‐up effects), rather than by the indirect effects of nutrients on its host's immune responses (top‐down effects).

### 4.1 In vivo bacterial performance

When larvae were sampled for bacteria during the exponential phase of bacterial growth in the host, the concentration of *X. nematophila* cells in the haemolymph was strongly negatively correlated with the amount of protein in the host diet and weakly positively correlated with the amount of dietary carbohydrate (Figure 3). This is, in part, consistent with a previous study using the same system and just six chemically‐defined diets, indicating that protein in the host diet reduced in vivo bacterial growth rate (Wilson et al., 2020). However, using 20 diets in this study allowed us to also detect the positive effect of dietary carbohydrate on bacterial performance, which was not observed in the six diets experiment (Wilson et al., 2020), suggesting that carbohydrates or their metabolites are being used by the bacteria as a food source. We also observed that the speed at which larvae succumbed to *X. nematophila* infection was strongly negatively related to the amount of protein in the host diet, and putative levels of protein in the larval haemolymph (Figure 4a). This is likely due to the bacterial population growing at a slower rate when the larvae ate protein‐rich diets and, as a consequence, had high levels of protein in the haemolymph (Holdbrook, Andongma, et al., 2024; Wilson et al., 2020).

This observed ‘protein effect’ could be due to a negative impact of the host's immune response on the in vivo bacterial growth rate. Consistent with this, a previous study using this system revealed that dietary protein facilitates the functional immune responses of *S. littoralis* against a *X. nematophila* challenge, but explains less than 12% of the variation in the immune response (Cotter et al., 2019). Disentangling these top‐down immune responses from bottom‐up resource utilization effects is challenging in host–parasite interactions but has been attempted in a number of systems (Griffiths et al., 2015; Haydon et al., 2003; Metcalf et al., 2011; Mideo & Reece, 2012; Moore et al., 2018; Ramiro et al., 2016), providing evidence for both top‐down and bottom‐up effects. For example, Metcalf et al. (2011) used a parameterised mathematical model to distinguish these effects in a mouse‐malaria host–parasite system. However, such an approach requires fine‐resolution time‐series data on the relative proportions of infected and non‐infected cells and is not appropriate for an extra‐cellular parasite like *X. nematophila*. A similar approach was taken by Moore et al. (2018) to explore the viral dynamics in humans vaccinated with the attenuated live virus that causes yellow fever. As with the mouse‐malaria model, however, these infections are long‐lasting and require repeated sampling of individuals over time, which is logistically challenging for short‐lived infections in short‐lived insects.

An alternative approach to establishing the relative importance of top‐down immune regulation and bottom‐up resource utilization is to remove the effects of the host immune response by quantifying in vitro parasite growth dynamics. Although previous studies have compared the effects of different nutrients on in vitro microbial growth, these usually involve batch cultures with generic media containing multiple nutrients that are simultaneously varied or the modification of a single dietary component (Bowen et al., 2012; Kooliyottil et al., 2014; Pulkkinen et al., 2018). In contrast, in the present study, we systematically varied key nutrients in 20 solutions that mimicked the nutritional conditions that the bacterium could expect to face within its host following different dietary regimes (Holdbrook, Randall, et al., 2024).

### 4.2 In vitro bacterial performance

For *S. littoralis* larvae fed on the 20 chemically‐defined diets, the haemolymph protein and carbohydrate levels are strongly negatively correlated with each other, making it difficult to distinguish their independent effects. However, the in vitro bacterial growth experiment clearly shows that bacteria grow best in nutribloods rich in carbohydrates and low in proteins (Figure 4b). When we compared this pattern of in vitro growth with that observed in vivo, the patterns were broadly similar, with both exhibiting a strong negative ‘protein effect’ (cf. Figure 4a,b). Moreover, when the standardized growth patterns were compared statistically, there was no overall detectable difference between the two (Figure 4c). This finding is consistent with bacterial growth largely being regulated by bottom‐up effects of the haemolymph nutrients, especially haemolymph protein, rather than top‐down effects of host nutrition on its immune response, as reflected in their relatively weak functional immune response to *X. nematophila* infection (Cotter et al., 2019). A number of studies have used in vitro studies of bacteria to predict growth properties in vivo, for example in the screening of potential probiotic bacteria (Foligne et al., 2007; Grangette et al., 2005) or antibiotics (Ono et al., 1996; Schmidtchen & Puthia, 2022). However, as far as we are aware, this is the first time that in vitro and in vivo approaches have been combined to study the effects of nutrition on a host–parasite interaction.

### 4.3 Proteins may interfere with osmoregulation

Abisgold and Simpson (1987) found that increasing protein concentration in the diet of *Locusta migratoria* increased haemolymph amino acid concentration, which in turn raised haemolymph osmolality. Osmoregulation, or a cell's ability to adapt to changes in their osmotic environment, is important for the maintenance of turgor pressure across the cellular membrane (Csonka & Hanson, 1991; Kempf & Bremer, 1998). The osmoregulatory ability of a cell, in turn, determines its ability to counteract osmotic stress and therefore its capacity to proliferate (Csonka, 1989; Tempest et al., 1970). The findings of Abisgold and Simpson (1987) highlight changes in osmolality as a possible mechanism for the observed ‘protein effect’. Indeed, Wilson et al. (2020) observed that the osmolality of *S. littoralis* haemolymph increased as the amount of protein in the host diet increased. Moreover, both in vitro and in vivo studies showed that *X. nematophila* growth rate declines with increasing osmolality, providing a potential mechanism for the observed negative effect of host dietary protein on bacterial growth and its positive effect on host survival (see fig. 5 in Wilson et al., 2020). It is pertinent to note that many animals self‐medicate in response to infection (e.g. Abbott, 2014; Erler et al., 2024). For example, the *S. littoralis* congener, *Spodoptera exempta* caterpillars increase their intake of dietary protein in response to bacterial infection (Povey et al., 2009) consistent with them triggering the negative ‘protein effect’ observed in this study.

## 5 CONCLUSIONS

The aim of this study was to use an in vitro system to determine whether the effects of host nutrition on a pathogen's growth could be due to direct (bottom‐up) effects in addition to the previously observed host‐mediated (top‐down) immunological effects (Cotter et al., 2011, 2019). We provide strong evidence that bacterial growth is primarily regulated by the bottom‐up effects of host nutrition, particularly via the negative effects of haemolymph protein, most likely via inducing osmotic stress on the bacterial cells (Wilson et al., 2020). Moreover, via the creation of synthetic insect haemolymphs (Holdbrook, Randall, et al., 2024), we provide a tractable experimental framework for testing the role of nutrition in host‐pathogen and host‐commensal relationships in insect blood. For example, one potential use of this system is to elucidate the nutritional requirements of the nematode symbionts, such as *S. carpocapsae*, that currently remain unknown (Richards & Goodrich‐Blair, 2009).

## AUTHOR CONTRIBUTIONS

Robert Holdbrook, Kenneth Wilson, Sheena C. Cotter, Stephen J. Simpson and Judith A. Smith conceived the ideas and designed methodology. Robert Holdbrook, Catherine E. Reavey, Joanna L. Randall, Yamini Tummala, Awawing A. Andongama and Annabel Rice collected the data. Robert Holdbrook, Kenneth Wilson and Sheena C. Cotter analysed the data. Robert Holdbrook, Kenneth Wilson and Sheena C. Cotter led the writing of the drafts. All authors contributed critically to the drafts and gave final approval for publication.

## CONFLICT OF INTEREST STATEMENT

The authors declare no conflicts of interest.

## Supporting information


**Figure S1.** The macronutrient composition of plants typically fed on by the generalist caterpillar, *Spodoptera littoralis*.
**Figure S2.** Effects of diet on standardised speed of death in *Xenorhabdus nematophila*‐challenged insects.
**Table S1.** Twenty diets fed to *Spodoptera littoralis* caterpillars varying in their ratios and concentrations of protein and carbohydrate.

## Data Availability

Data available from the Dryad Digital Repository: https://doi.org/10.5061/dryad.pzgmsbcxv (Wilson et al., 2024).
