# Supplementary material for: Combining in vivo and in vitro approaches to better understand host‐pathogen nutritional interactions
Source: J Anim Ecol. 2025 Feb 7;94(4):657–69. doi: 10.1111/1365-2656.70000 (PMC11962230; doi:10.1111/1365-2656.70000)
Supplement: Supplementary file 1 — Figure S1. The macronutrient composition of plants typically fed on by the generalist caterpillar, Spodoptera littoralis. Figure S2. Effects of diet on standardised speed of death in Xenorhabdus nematophila‐challenged insects. Table S1. Twenty diets fed to Spodoptera littoralis caterpillars varying in their ratios and concentrations of protein and carbohydrate. [file JANE-94-657-s001.docx]

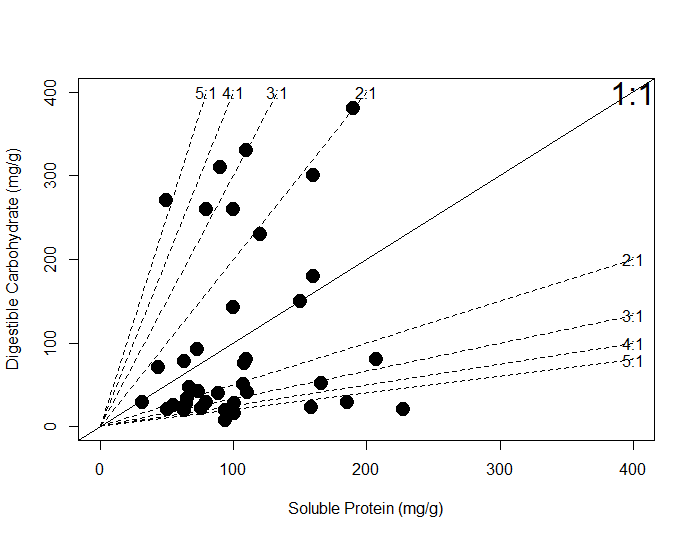


**Figure S1. The macronutrient composition of plants typically fed on by the generalist caterpillar, *S. littoralis*.** The data were taken from Scott Brown et al. (2002) and J. K. Wilson et al. (2019).


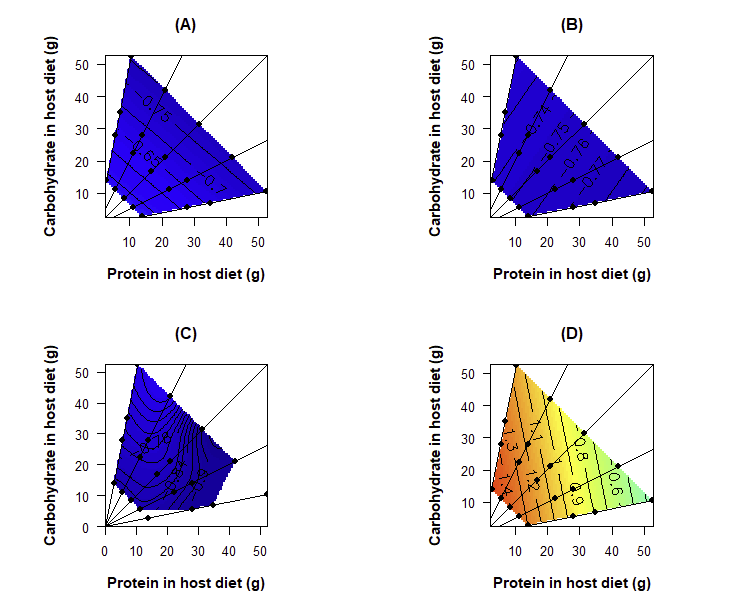


**Figure S2. Effects of diet on standardised speed of death in *X. nematophila*-challenged insects.** (A) Challenged with dead bacteria; (B) sham-challenged; (C) challenged with live bacteria and survived; (D) challenged with live bacteria and died.

## Table S1: Twenty diets fed to *Spodoptera littoralis* caterpillars varying in their ratios and concentrations of protein and carbohydrate. For full details of the diets used see Table S1 in Cotter et al (2019).

| **Diet** | **P:C ratio** | **Diet concentration**  **(g/100g)** | **% protein** | **Protein (g/100g)** | **Carbohydrates (g/100g)** |
| --- | --- | --- | --- | --- | --- |
| 1 | 1:5 | 63 | 17 | 10.5 | 52.5 |
| 2 | 1:5 | 42 | 17 | 7 | 35 |
| 3 | 1:5 | 33.6 | 17 | 5.6 | 28 |
| 4 | 1:5 | 16.8 | 17 | 2.8 | 14 |
| 5 | 1:2 | 63 | 33 | 21 | 42 |
| 6 | 1:2 | 42 | 33 | 14 | 28 |
| 7 | 1:2 | 33.6 | 33 | 11.2 | 22.4 |
| 8 | 1:2 | 16.8 | 33 | 5.6 | 11.2 |
| 9 | 1:1 | 63 | 50 | 31.5 | 31.5 |
| 10 | 1:1 | 42 | 50 | 21 | 21 |
| 11 | 1:1 | 33.6 | 50 | 16.8 | 16.8 |
| 12 | 1:1 | 16.8 | 50 | 8.4 | 8.4 |
| 13 | 2:1 | 63 | 67 | 42 | 21 |
| 14 | 2:1 | 42 | 67 | 28 | 14 |
| 15 | 2:1 | 33.6 | 67 | 22.4 | 11.2 |
| 16 | 2:1 | 16.8 | 67 | 11.2 | 5.6 |
| 17 | 5:1 | 63 | 83 | 52.5 | 10.5 |
| 18 | 5:1 | 42 | 83 | 35 | 7 |
| 19 | 5:1 | 33.6 | 83 | 28 | 5.6 |
| 20 | 5:1 | 16.8 | 83 | 14 | 2.8 |
